# Tumor Microenvironment Autophagic Processes and Cachexia: The Missing Link?

**DOI:** 10.3389/fonc.2020.617109

**Published:** 2021-02-02

**Authors:** Renata de Castro Gonçalves, Paula Paccielli Freire, Dario Coletti, Marilia Seelaender

**Affiliations:** ^1^ Cancer Metabolism Research Group, Department of Surgery, LIM26-HC, Faculdade de Medicina, and Institute of Biomedical Sciences, University of São Paulo, São Paulo, Brazil; ^2^ Department of Immunology, Institute of Biomedical Sciences, University of São Paulo, São Paulo, Brazil; ^3^ Sorbonne Université, CNRS UMR 8256, Inserm U1164, Biological Adaptation and Aging (B2A), Paris, France; ^4^ Department of Anatomy, Histology, Forensic Medicine & Orthopedics, Histology & Medical Embryology Section, Sapienza University of Rome, Rome, Italy

**Keywords:** cachexia, autophagy, metabolism, DAMPs, lymphocyte infiltration, tumor microenvironment, systemic inflammations

## Abstract

Cachexia is a syndrome that affects the entire organism and presents a variable plethora of symptoms in patients, always associated with continuous and involuntary degradation of skeletal muscle mass and function loss. In cancer, this syndrome occurs in 50% of all patients, while prevalence increases to 80% as the disease worsens, reducing quality of life, treatment tolerance, therapeutic response, and survival. Both chronic systemic inflammation and immunosuppression, paradoxically, correspond to important features in cachexia patients. Systemic inflammation in cachexia is fueled by the interaction between tumor and peripheral tissues with significant involvement of infiltrating immune cells, both in the peripheral tissues and in the tumor itself. Autophagy, as a process of regulating cellular metabolism and homeostasis, can interfere with the metabolic profile in the tumor microenvironment. Under a scenario of balanced autophagy in the tumor microenvironment, the infiltrating immune cells control cytokine production and secretion. On the other hand, when autophagy is unbalanced or dysfunctional within the tumor microenvironment, there is an impairment in the regulation of immune cell’s inflammatory phenotype. The inflammatory phenotype upregulates metabolic consumption and cytokine production, not only in the tumor microenvironment but also in other tissues and organs of the host. We propose that cachexia-related chronic inflammation can be, at least, partly associated with the failure of autophagic processes in tumor cells. Autophagy endangers tumor cell viability by producing immunogenic tumor antigens, thus eliciting the immune response necessary to counteract tumor progression, while preventing the establishment of inflammation, a hallmark of cachexia. Comprehensive understanding of this complex functional dichotomy may enhance cancer treatment response and prevent/mitigate cancer cachexia. This review summarizes the recent available literature regarding the role of autophagy within the tumor microenvironment and the consequences eliciting the development of cancer cachexia.

## Introduction

Cachexia is a multifactorial syndrome characterized by inducing continuous and involuntary weight loss (1). This complex comorbidity occurs in association with malignant disease and with various other chronic diseases. Cancer cachexia consists of chronic systemic inflammation and catabolism, decreasing treatment response and survival (2). The most pronounced symptoms of cachexia syndrome can include energy imbalance and immune system impairment, anorexia, neuroendocrine changes, asthenia, nausea, malabsorption, but by far the most representative symptom is body mass weight loss, due to both fat and lean mass wasting (3). Cachexia affects up to 80% of advanced cancer patients and is highly associated with specific tumor types such as pancreatic, esophageal, gastric, lung, and liver (4–6). The etiology of cancer cachexia in different tumor types involves complex and specific tumor–host interactions that remain to be completely elucidated.

Several factors contribute to inflammation in cancer cachexia, as eicosanoids (7), augmented fatty acid content in the circulation (8), and increased circulation of pro-inflammatory cytokines, among which tumor necrosis factor (TNF), interferon (IFN), interleukin-6 (IL6), and interleukin 1 beta (IL1B) play a prominent role (9). Different tissues and cells can secrete cachexia-inducing factors (10). Therefore, depending on the percentage of infiltrating immune cells, the tumor microenvironment may have a different inflammatory infiltrate profile, accompanied by a specific secretion profile. In fact, non-neoplastic cells within the tumor microenvironment and surroundings, including immune cells fibroblasts and endothelial cells, play an important role in regulating the secretion of cachexia-inducing factors by the tumor (11). According to this, we have previously demonstrated that pro-inflammatory cytokines in the tumor microenvironment generate chronic local inflammation, stress, and influencing the progression of cachexia (12).

Derived from the Greek, autophagy “self-digestion” refers to a cellular lysosomal degradation pathway (13). Autophagy can be non-selective (such as during nutrient deprivation) or selective (such as, when damaged organelles or intracellular pathogens are broken up) (14). This pathway involves forming double-membraned vacuoles called autophagosomes, which sequester cytoplasmic material delivered to the lysosome to be degraded and recycled (14). Autophagy is important for regulating cellular metabolism and homeostasis. Under physiological conditions, it is a conservative process that controls cellular balance, maintains homeostasis (15), and ensures proper energy metabolism, by preventing small detrimental energy status variations. In the absence of stimuli, basal autophagy, by eliminating unnecessary organelles and non-useful aggregate proteins, allows normal renewal of proteins and organelles, thus enabling cells to generate energy when nutrients are scarce, and providing bioenergetic support during development (survival response) (15). Under pathological conditions, autophagy elicits adaptive responses, to stress that damages the endoplasmic reticulum or to metabolic alterations. The cytoplasmic organelle that should be eliminated is explicitly identified by the autophagic machine and eliminated through a mechanism described as “selective autophagy” (15). There are several forms of stress-induced autophagy: adenosine diphosphate (ADP) accumulation, increased concentration of reactive oxygen species (ROS), inflammation, hypoxia, and endoplasmatic reticulum stress (ER stress).

Oxidative stress has a crucial role in autophagy induction. Healthy mitochondria generate ROS by regularly oxidative phosphorylation, but under stressful circumstances, damaged mitochondria can produce high ROS levels as to induce mitochondrial biogenesis or mitophagy creating a redox negative feedback loop (16). Among the multiple cellular effects of ROS (17) Tang et al., have found that ROS promotes mild oxidation on High-mobility group box-1 (HMGB1), which leads to its translocation into the cytosol and induces cellular autophagy by enhancing ERK (extracellular signal-regulated kinase) signaling, and disrupting beclin1–Bcl-2 complex formation (17). Therefore, autophagic process represents an anti-inflammatory mechanism of protection against endomembrane damage (18) and cellular stress. Thus, defective autophagy can contribute to establishing an inflammatory microenvironment (19).

## Autophagy Role in Inflammation And Immune Response Control

Autophagy is a process that acts on a two-way road during tumorigenesis and tumor progression. The process of autophagy is an important mechanism for tumor-suppression during the early stages of cancer, preventing inflammation and genome instability (20); yet, it can paradoxically improve cancer cell survival during transformation-induced metabolic stress (21). Autophagy-related tumor suppression occurs mainly by preventing ROS accumulation by removing damaged mitochondria, inhibiting deleterious effects on DNA mutation, which may induce tumorigenesis (22).

Lindsay DeVorkin et al. (23), demonstrated that tumors from Atg5^−^/^−^ mice (an autophagy-deficient model) present a reduction in the number of tumor-infiltrating T cells (TILs) CD8 T cells. The autophagic process delivers cytoplasmic components to lysosomes, thus contributing to cytoplasmic immune recognition and response. Antigen processing and presentation are regulated by autophagy-related genes, thus influencing innate and adaptive immunity (24). Nanoparticles of *α*-alunima (*α*-Al2O3-autophagy inducer) increase the delivery of antigens to dendritic cells (DCs) in autophagosomes, contributing to tumor regression (25). Cross-presentation of tumor-associated antigens by MHC class I molecules, an important feature of antitumor immune response, is regulated by autophagy-mediated lysosomal proteolysis and proteasomal degradation (26).

The tumor microenvironment comprises various types of stromal cells, including fibroblasts, vascular endothelial cells, immune cells, adipocytes, mesenchymal stem cells (MSCs), and cytokines are present at high concentrations in this *milleu* (27). Within this complex cell population, the response to multiple metabolic stressors activates autophagy, which may impair anticancer immune response and allow tumor cells to evade immune surveillance, resulting in tumor growth and cancer progression (28). In the study by Lindsay DeVorkin and coll. (23), the Atg5^−^/^−^ CD8+ T cells were shown to display enhanced glucose metabolism, which increases tri-methylation on histone H3 lysine 4 (H3K4me3) density, hence affecting the transcriptional upregulation of both effector and metabolic target genes. These findings identify autophagy as a negative regulator of CD8+ T cell metabolism and, consequently, antitumor immunity, with implications for T cell-based immunotherapy (23).

Cancer-associated fibroblasts (CAFs) and cancer-associated adipocytes (CAAs) exhibit increased autophagic flux, as compared to their respective normal phenotypes, in part as a consequence of local hypoxia-associated ROS production by malignant cells, transforming growth factor-beta 1 (TGF-*β*1) signaling and, extracellular matrix remodeling (29). Tumor microenvironment autophagy in endothelial and stromal cells influences disease progression and response to treatment since it supports tumor progression through the secretion of mitogenic cytokines, such as interleukin-6 (IL6) and interleukin-8 (CXCL8) (29). The increased expression of IL6 and CXCL8 has also been strongly associated with the development of systemic inflammation observed in patients with cachexia (30–32). These cytokines help to recruit the metabolic substrates to neoplastic cells to fuel oxidative phosphorylation, including fatty acids, ketone bodies, alanine, and lactate (29).

Autophagy and ROS generation in adjacent CAFs induced by altered signaling pathways in cancer cells can explain the mechanistic association between tumor microenvironment and cancer cachexia (33). It is worth to mention that cancer cachexia shares similarities with stromal-carcinoma metabolic synergy theory, given its main characteristic of wasting both fat and lean mass (34). The authors have pointed that autophagy induced in the tumor stroma can be homologous (but in a smaller proportion) with systemic wasting in the cancer-associated cachexia (35). In this study, the authors have explained that cancer cachexia may start locally as stromal autophagy and consequently spread systemically. As such, the authors suggested that stromal autophagy may be the essential precursor of the cachexia syndrome (35).

**Figure 1:**
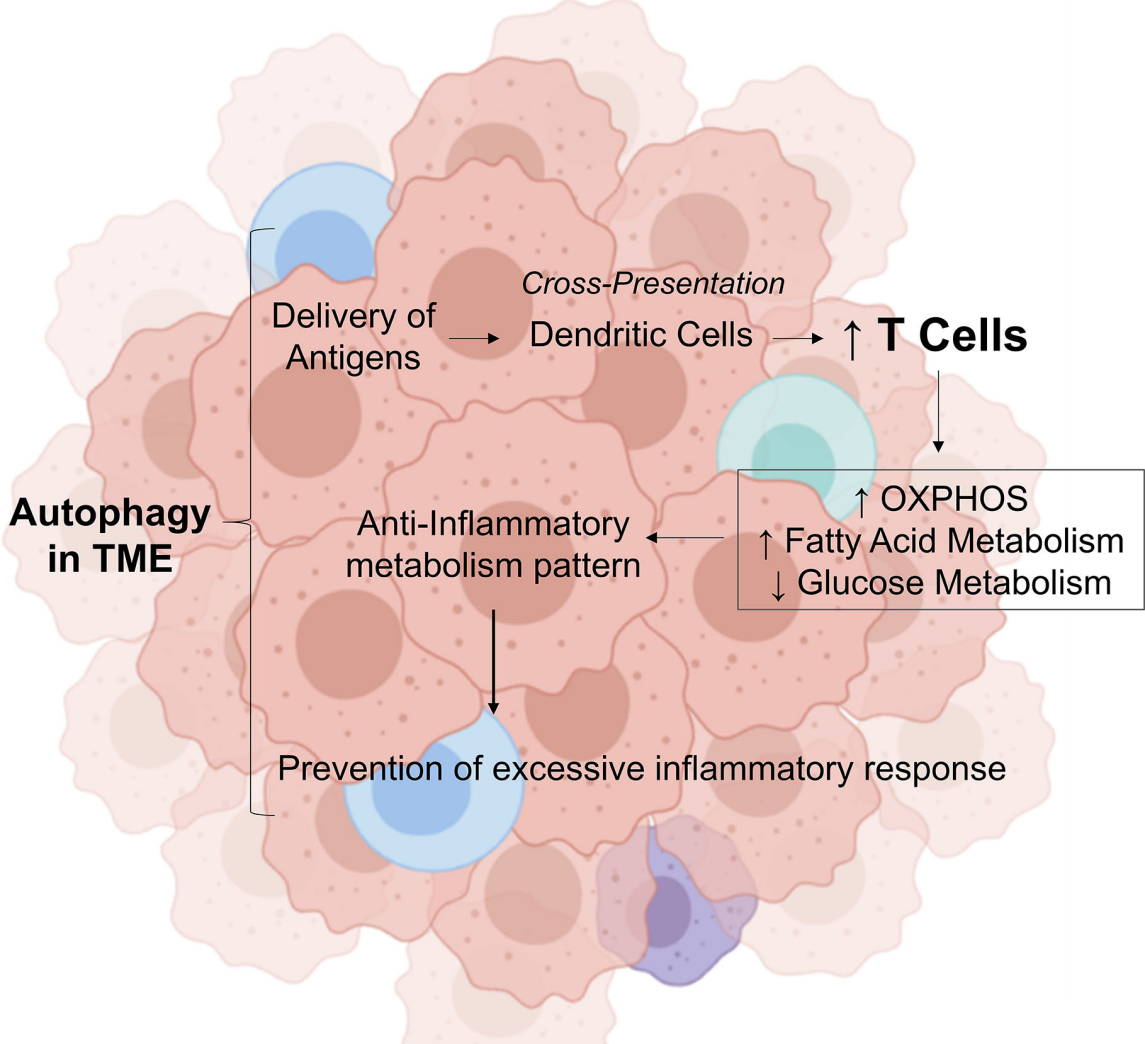
Role of autophagy in tumor microenvironment in modulating the inflammatory response. In the tumor microenvironment, autophagy provides the delivery of antigens. In this environment, antibodies attach to these antigens and attract immune cells by dendritic cells. This process stimulate the increase of CD8 T cells, which induces a metabolic shift by decreasing the glucose metabolism, increasing the fatty acid oxidation and OXPHOS. This transition from glycolytic metabolism is important to control cytokine production, then preventing excessive inflammatory response. OXPHO, Oxidative Phosphorylation; TME, Tumor Microenvironment; Credit, Created with BioRender (https://biorender.com/).

These findings demonstrate that autophagy is crucial for the prevention of tumorigenesis and for the initiation of antitumor immune response; however, paradoxically, chronic sustained autophagy can affect tumor infiltrated immune cells metabolism, increasing fatty acid oxidation (FAO), thereby acting as a negative regulator of antitumor immune response, and preventing excessive inflammation and tissue damage in the tumor tissue, in addition, to promote metabolic support for the survival of cancer cells through the induction of autophagy in tumor-associated stromal cells (**Figure 1**).

Both chronic infection and cancer require the ability of lymphocytes to sustain cell function despite persistent antigen stimulation. However, in a context of self-antigens or excessively damage healthy tissue target, maintaining robust T cells response can prove harmful rather than helpful; therefore, T cell exhaustion represents a critical mechanism through which CD8+ T cells lose effector ability owing to persistent stimulation (36). It is important to note that effector lymphocyte exhaustion not only curtails the ability to cause damaging immunopathology but as a consequence, facilitates viral persistence and hampers tumor-targeted responses (36).

Considering that nutrients availability is essential for controlled cellular proliferation, growth, and survival in organisms and that cells continuously sense environmental changes and adapt to stress signals, we can conclude that maintenance of the autophagic flux, a mechanism of cellular survival, in immune cells in tumor microenvironment conditions is essential for the maintenance of host homeostasis.

## Systemic Consequences of Autophagic Process: Effect on Muscle and Fat Tissues

The depletion of skeletal muscle is a key feature of cancer cachexia (1). Studies in tumor-bearing animals demonstrated that accelerated proteolysis, primarily by the ubiquitin-proteasome system, and several clinical studies also describe a substantial decrease in muscle mass (37). It is very likely that systemic inflammation could play a pivotal role in cachexia- associated muscle wasting. We, therefore, need to recognize the origin of systemic inflammation in cancer patients to seek new, more effective therapies for counteracting cachexia.

The maintenance of hypoxia-induced stress in the tumor microenvironment mimics tissue damage (in the case of solid tumors), and this can trigger Damage-associated molecular patterns/toll-like receptor (DAMP/TLR)-dependent inflammatory responses, even without overt tissue damage (38). This cancer-associated tissue injury is perpetuated by the homeostatic inflammatory and tissue repair responses (38), leading to High Mobility Group Box 1 (HMGB1) release, being this the most abundant DAMP released by the stressed tumor cells (39). Altered HMGB1 redox state plays a dynamic role in malignancies, binding to toll-like receptors TLR2, TLR4, and TLR9 (39). In line with this, Zhang et al. (40) have demonstrated that in the cancer cachexia model of Lewis Lung Carcinoma (LLC) muscle catabolism is directly activated by activation of TLR4 in the fibers. In LLC tumor-bearing mice the increase of circulating TNFα and IL6 depends on TLR4. These data suggest that tumor-induced activation of TLR4 is responsible for both protein catabolism in muscle cells and systemic inflammation, suggesting that anti-TLR4 strategies could achieve interesting results (40). Moreover, significant HMGB1 protein release is associated with poor prognosis (41). These studies demonstrate that danger-associated molecular patterns (DAMPs) act as catabolic extracellular signals and are linked to worsened prognosis in cancer patients.

In a mouse model of colorectal carcinogenesis, Yi Luo et al. (42) examined the effects of muscle HMGB1 on pyruvate kinase status and autophagy-proteolysis in muscle. The study demonstrates that HMGB1 secretion induced by autophagy in the tumor microenvironment during tumorigenesis can alter the plasma levels of glutamine, which is utilized as an energy source by cancer cells, and decrease pyruvate kinase isozymes M1 (PKM1). The authors also suggest that HMGB1 released by the tumor helps to recruit glutamine from the muscle to supply cancer cells as an energy source. Together, these results demonstrate that the stressed tumor microenvironment modulates an inflammatory antitumor immune response. However, this response seems to be linked to cachexia’s characteristics associated with cancer and, mainly, with poor prognosis.

A Pan-Cancer analysis using 21 tumor types from The Cancer Genome Altas (TCGA) demonstrated that the profile of tumor purity (proportion of tumor cells and non-neoplastic cells in the heterogeneous mixture) differs according to tumor type and is correlated with patient prognosis (43). Another extensive immunogenomic study of more than 10,000 tumors from TCGA demonstrated that patients with tumor types with IFN-*γ* dominant phenotype showed a less favorable survival, despite tumor higher lymphocytic infiltrate, a CD8 T cell-associated signature, and higher M1 content (44). Interestingly, pancreatic adenocarcinoma, known to have the highest prevalence of cachexia (2), is associated with low purity tumors and is characterized by low neoplastic cellularity and high leukocyte fraction (44, 45). In this line, a recent study demonstrated high expression of cachexia-inducing factors, such as CXCL8, IL6, IL1B, CCL2, and TNF, in pancreatic cancer patients, whose tumor was classified as of low-purity (higher proportion of immune cells than of malignant cells) (46). These results also suggest that infiltrating immune cells are the main contributing cells to the cachectic tumor phenotype.

**Figure 2:**
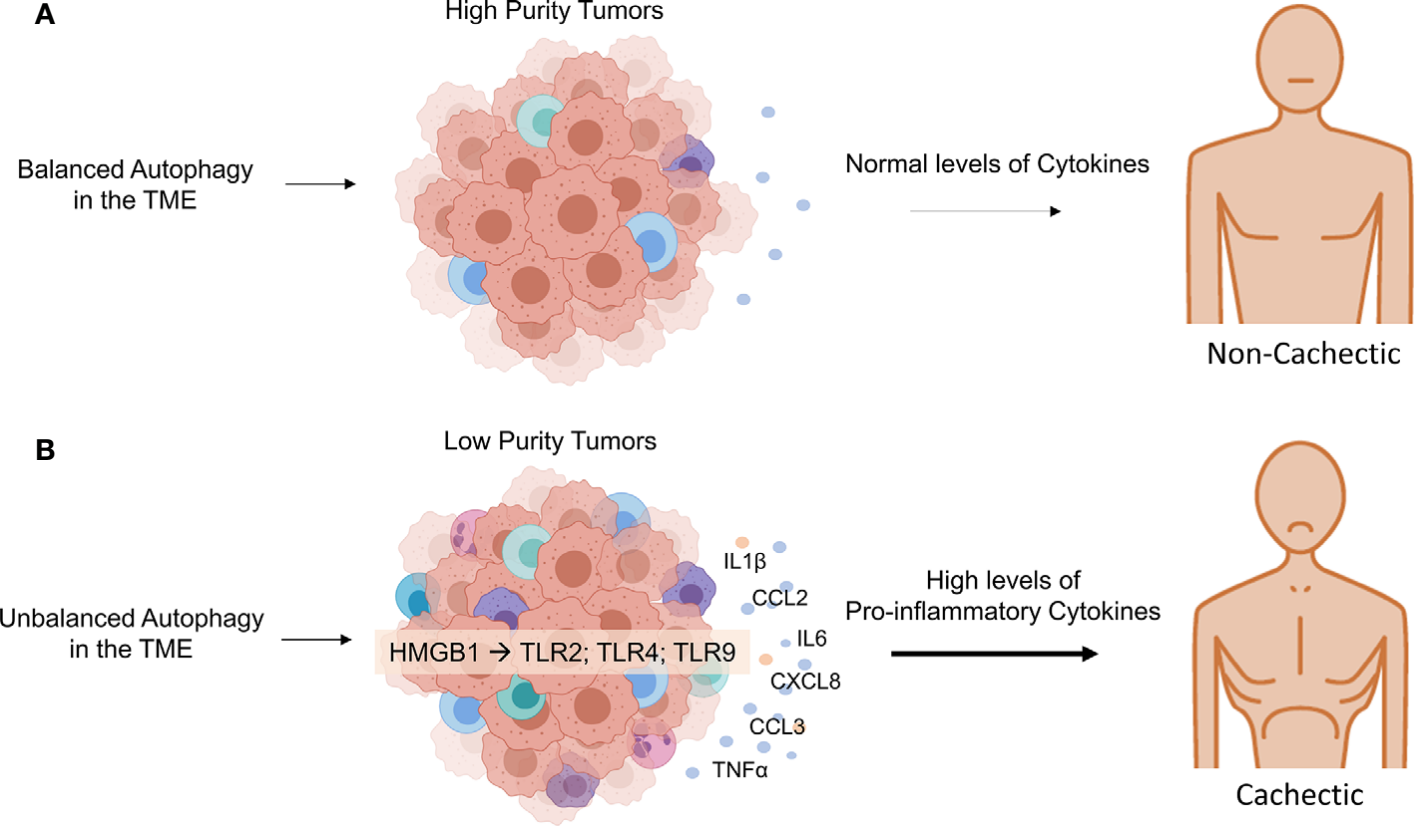
The systemic effect of autophagy balance in tumor microenvironment. **(A)** In a tumor microenvironment with balanced autophagy, there is tumor immunogenicity, which explains the characteristics of tumors classified with high purity. Tumors with small proportions of immune cells have a controlled inflammatory response and, therefore normal production of proinflammatory cytokine levels in cancer patients. **(B)** The effect of unbalanced autophagy in the tumor microenvironment, attracting immune cells into the tumor environment, result from HMGB1 acting on TLRs in immune cells. Unbalanced autophagy in the TME can lead to non-functional control of immune cells’ inflammatory phenotype that can upregulate metabolic consumption and cytokine production in the tumor microenvironment and systemic levels in the host, an important feature of cancer cachexia. Credit: Created with BioRender (https://biorender.com/).

Moreover, it has been recently demonstrated that tumors from cachectic patients have higher TNF-α and monocyte chemoattractant protein 2 (CCL2) gene expression, along with higher CCL3 protein expression than those of weight stable cancer patients (12). Inflammation-related factors in whole tumor samples were shown to present distinctive expression of pro-inflammatory cytokine as IL-1β and anti-inflammatory cytokine as IL13 (higher and lower, respectively) in cachectic cancer patients and weight stable counterparts. Finally, lower numbers of M2 macrophages in cachectic cancer patients’ tumors were found, as compared with the weight stable group. These results demonstrate that cachectic patients’ tumor microenvironment seems to direct the infiltrating immune cells to a more inflammatory phenotype (**Figure 2**).

In colon cancer, Yihao Mao et al. (11) have demonstrated that low tumor purity is associated not only with poor prognosis but also with immunotherapy-associated markers, such as PD-1 (Programmed cell death protein 1), PD-L1 (Programmed cell death ligand-protein 1), CTLA-4 (cytotoxic T-lymphocyte-associated protein 4), LAG-3 (Lymphocyte-activation gene 3), and TIM-3 (T cell immunoglobulin- and mucin-domain-containing molecule-3). These findings indicate exhaustion of the immune response within the tumor microenvironment. The same authors also showed that the mutation burden in low purity colon cancer was significantly heavier than that of high purity tumors (11).

It is known that immune cells adopt a range of functional profiles depending on the environment. Lymphocytes rapidly mount an effector response to inflammatory signals (47). Induction of *de novo* fatty acid synthesis is essential for activation-induced proliferation and differentiation of effector T cells, predominantly glycolytic metabolism (48) in addition to enhanced glucose and glutamine catabolism to rapidly generate energy and feed anabolic pathways (47). It is important to note that one of the cachexia features is increased resting energy expenditure, yielding negative energy balance. Tumors and tumor-associated cells compete with other organs and tissues for energy fuels and biosynthetic substrates and possess an intrinsic metabolic rate related to their mass and degree of aerobic versus anaerobic energy metabolism (2).

Thomas Riffelmacher et al., demonstrated the role of ATG proteins in restricting inflammation *via* LC3-associated phagocytosis (LAP) and alterations of cytokine release in macrophages (49). A possible explanation the direct link of autophagy-dependent metabolism and cellular differentiation, occurring by decreased monocytes’ potential differentiation into M2 macrophages in atg7-KO mice, along with increased glycolytic activity, M1 pro-inflammatory cytokine production, and reactive oxygen species production (49).

Based on the aforementioned knowledge, we hypothesize that maintenance of hypoxia-induced stress within the tumor microenvironment mimicking tissue damage induces the release of catabolic extracellular signals that attract immune cells into the tumor environment. Consequently, a defective autophagic regulation induces a pro-inflammatory tumor microenvironment, modulating the catabolic host state and the loss of muscle and adipose tissue in cachectic cancer patients.

In conclusion, autophagy appears to play a balancing act in supporting productive inflammatory response while simultaneously preventing excessive inflammatory and tissue damage, acting as a process to maintain host metabolic homeostasis, preventing cancer cachexia. When this fails, cachexia appears.

Mechanistic approaches are essential for the advancement of research in the study of cancer and for the development of drugs acting in certain phases of tumorigenesis. Understanding the nature of progressive malignancy diagnosis and more accurate and appropriate treatment for each case. In this review, we hypothesized that tumor progression and cachexia are partly sustained by the paradoxical effect of autophagy in the tumor microenvironment.

## Author Contributions

RG is the principal writer. PF is the writer and corrected the manuscript. DC was in charge of the current literature and corrections. MS is the supervisor. All authors contributed to the article and approved the submitted version.

## Conflict of Interest

The authors declare that the research was conducted in the absence of any commercial or financial relationships that could be construed as a potential conflict of interest.
